# Effectiveness of an adapted physical activity intervention on health-related physical fitness in adolescents with intellectual disability: a randomized controlled trial

**DOI:** 10.1038/s41598-022-26024-1

**Published:** 2022-12-30

**Authors:** Yan Sun, Siyue Yu, Aiwei Wang, Hardaway Chun-Kwan Chan, Alison Xiaoting Ou, Dexing Zhang, Yaojie Xie, Shirley S. M. Fong, Yang Gao

**Affiliations:** 1 Department of Sport, Physical Education and Health, Hong Kong Baptist University, Hong Kong, China; 2 JC School of Public Health and Primary Care, The Chinese University of Hong Kong, Hong Kong, China; 3 Scientific Conditioning Centre, Hong Kong Sports Institute, Hong Kong, China; 4 School of Nursing, The Hong Kong Polytechnic University, Hong Kong, China; 5 School of Public Health, University of Hong Kong, Hong Kong, China; 6 Department of Health and Physical Education, Education University of Hong Kong, Hong Kong, China; 7 Centre for Health and Exercise Science Research, Hong Kong Baptist University, Hong Kong, China

**Keywords:** Risk factors, Neurological disorders, Clinical trial design, Paediatrics, Public health, Weight management

## Abstract

This study aimed to examine the effects of an adapted physical activity (APA) intervention on health-related physical fitness (HRPF) in adolescents with intellectual disability (ID). With a randomized controlled trial design, adolescents aged 12–18 years, with mild and moderate ID, and being overweight and obese were recruited and randomly assigned to either an intervention or a control group. The APA intervention consisted of overall moderate intensity aerobic and resistance exercise, with a duration of 45 min/session, a frequency of 2 sessions/week, and lasting for 9 months. A negative control was involved, in which participants received no treatment. Changes in four fitness tests, including the 9-min run/walk, handgrip strength, 30-s sit-ups, and sit-and-reach tests, were assessed between the groups using general linear models. A total of 57 participants (39 in the intervention group and 18 in the control group) completed the study. Significant mean differences in changes in the 9-min run/walk test (413.6 m [95% CI 146.72 m, 680.41 m], *p* = 0.003) and the right-side sit-and-reach test (2.2 cm [95% CI 0.37 cm, 4.09 cm], *p* = 0.020) respectively were observed in the intervention group, compared to the control group. No significant between-group improvement was observed for the handgrip strength and the 30-s sit-ups tests. The APA intervention induced beneficial effects on cardiorespiratory fitness and potential beneficial effects on flexibility for adolescents with ID. However, no significant effects of this intervention on muscular strength and endurance were observed in this study. Future studies should consider involving effective exercises in interventions to improve muscular strength and endurance.

## Introduction

In children and adolescents, a poor level of health-related physical fitness (HRPF) and related negative health-related outcomes may track into adulthood, which may also be a predictor of mortality and morbidity across the life span^1^. HRPF related benefits, for example, cardiorespiratory fitness and muscular fitness, are inversely associated with cardiovascular disease risk factors (e.g., lipids profile, blood pressure, body fat^2,3^); flexibility and muscular fitness are negatively associated with risks of injuries to lay a foundation for balance and motor skill development, and favourable to skeletal health^1,4^. In addition, they could reap benefits regarding mental well-being^1,5^ and health-related quality of life^6^ from better HRPF.

In comparison with the typically developing peers, the benefits of HRPF are even more critical for children and adolescents with ID, as they tend to have more physical health problems (such as diabetes and obesity), less developed motor skills, and more mental health problems^7–9^. It has been reported consistently that children and adolescents with intellectual disability (ID) performed much poorer in HRPF assessments, mainly due to the reduced physical activity (PA) level^10–12^. For example, a previous Dutch study suggested that children with ID performed lower levels of cardiorespiratory fitness and muscular strength than typically developing children, reporting medium and moderate to large effect sizes, respectively^10^. Another study identified 71–90% of children and adolescents with moderate to severe ID scored below the 5th percentile of reference values of typically developing peers in HRPF assessments^12^. However, evidence on HRPF levels was limited in children and adolescents with ID in Hong Kong^13,14^. Our previous cross-sectional study conducted on a sample of 524 children and adolescents with ID in Hong Kong, demonstrated that the combined prevalence of overweight and obesity was 31.3%, and 93.7% of them did not meet the global PA recommendation for at least 60 min of moderate-to-vigorous PA (MVPA) daily^15^. The findings of the study also indicated that adolescent children (aged 13–18 years) were more likely to be overweight, obese, and physically inactive than young children (aged 6–12 years)^15^. Participation in PA could strikingly enhance the HRPF levels, which will, in turn, enable children and adolescents to engage in a wider range of physical activities, further produce greater benefits to health^13^. Hence, there is an urgent need for PA interventions to improve HRPF among adolescents with ID, especially those with overweight and obesity.

Substantial evidence suggests that PA interventions have beneficial effects on physical fitness in typically developing adolescents^16,17^. However, PA intervention studies examining HRPF indicators in adolescents with ID are scanty. We recently conducted a systematic review and meta-analysis to identify effective intervention strategies for improving HRPF levels among this special paediatric population^18^. We found that most studies reviewed were non-randomized controlled trials (RCT), focusing on aerobic exercise and related effects on cardiorespiratory fitness indicators, and with a short-term duration (i.e., ≤ 3 months)^18^. In addition, there was a paucity of evidence to support other HRPF components (i.e., flexibility, muscular strength and endurance). Given that schools are ideal settings to deliver PA interventions for children and adolescents with ID, we therefore designed and conducted a 9-month school-based adapted PA (APA) intervention, which primarily aimed to reduce the body weight (primary outcomes); secondly aimed to enhance the levels of other HRPF components among adolescents with ID and overweight or obesity. We have reported that this APA intervention was effective in improving obesity-related indicators where else^19^. This paper presents the intervention effects on other HRPF parameters, including cardiorespiratory fitness, muscular strength and endurance, and flexibility.

## Methods

### Study design and participants

This APA intervention was a 9-month parallel-group RCT registered at Clinicaltrials.gov (NCT04463069; 09/07/2020). The trial was conducted among adolescents from six special schools in Hong Kong between October 2018 and June 2019. Participants were randomized 1:1 to the APA intervention group or the control group using block randomization. The random sequence was generated from a computerized random-number generator, by an off-site statistician. Participants were eligible for inclusion in this trial if they were adolescents (age range 12–18 years), overweight or obese (determined based on the specific cut-off points for age and sex established by Cole^20^), and with mild or moderate ID (i.e., intelligence quotient (IQ) range: 35–69). Participants were ineligible if they had a physical disability, contraindications to performing regular physical activities, or had attended other exercise programs in the previous six months. Participants who were prescribed medications that may influence weight status were also excluded from the study. School nurses identified eligible participants and invited them to participate. The study was approved by the Research Ethics Committee of Hong Kong Baptist University (HASC/17-18/0721), and conducted in accordance with the Declaration of Helsinki. Written informed consent was obtained from all participants’ parents/guardians prior to participating in the study.

### Intervention

The detailed description of the intervention has been described elsewhere^19^. In brief, this intervention was a school-based 9-month moderate intensity PA program modified from a previous APA program for adolescents with ID^21^. The intervention provided participants with a simple and joyful exercise regime at a frequency of two 45-min training sessions per week for three consecutive 3-month periods. Each training session began with a 5-min warm-up to improve body mobility, followed by a 35-min strength and endurance exercise, and ended with a 5-min cool-down which allowed for relaxation and stretching. The training/exercise protocol (as described in Table S1) was developed by the research team and the school physical education (PE) teachers. The intervention was conducted by research team members with assistance from school teachers in the school playground during school hours. The training intensity was gradually increased from 30–40% maximal heart rate reserve (% HRR) at Stage 1, to 40–50% HRR at Stage 2, and to 50–60% HRR at Stage 3. Participants were given 2–3 weeks at each stage to adapt to the increased training intensity. The participants’ real-time exercise heart rates (exercise HRs) during each session were monitored by instructors through counting radial pulses for 10 s and then multiplying the values obtained by six (beats/min) used to determine the training intensity of each individual, with measured HR at rest and estimated HRmax using the Fernhall’s equation^22^. The exercise HR was compared with the target exercise HR to determine the intensity compliance rate (%) defined as the percentage of participants in the intervention achieving target exercise HR. The stage attendance rates for each participant in the intervention were recorded by instructors.

#### Control

The participants allocated to the control group received no treatment during the study period.

## Measures

### Outcome measures

Outcome measures including HRPF components and tests were performed in the following sequence: muscular strength and endurance (measured by handgrip strength and 30-s sit-ups), flexibility (measured by sit and reach test) and cardiorespiratory fitness (measured by 9-min run/walk test)^23^. All tests were conducted following standard procedures/protocols at the pre-intervention assessment (pretest) and post-intervention assessment (posttest). All participants were introduced with the procedures and techniques in advance and received instructions during the tests.

#### Cardiorespiratory fitness

Cardiorespiratory fitness was assessed using the 9-min run/walk test that has been proved to be valid and reliable in adolescents^24^. Participants were encouraged to run and obtain the greatest possible distance on a flat surface 25 m in length over a 9-min period. Participants were encouraged to maintain a steady pace throughout the running test. The distance was recorded to the nearest meters.

#### Muscular strength and endurance

The handgrip strength test and 30-s sit-ups were used to measure muscular strength and endurance. The handgrip strength test was applied to measure the strength of the hand and forearm muscles, which was commonly used to indicate muscular strength^25,26^. The participants were instructed to maintain a standing position, with arms parallel but not touching the body, and to squeeze a handgrip dynamometer (Takei, TKK5001, GRIP-A digital dynamometer) as hard as possible. The participants were allowed a total of four attempts to squeeze the dynamometer with each hand using two attempts. A pause of 10–20 s was allowed between measurements to avoid the effects of muscular fatigue. All individual attempts were recorded and the better score of each hand was used for data analysis^10^. The 30-s sit-ups test was adopted to measure abdominal muscular strength and endurance. Participants were asked to lie down in a supine position with their knees bent at 90 degrees and heels and feet placed flat on the mat. They were required to cross both arms in front of their chests with each hand embracing the opposite shoulder. The test required the subjects to rise up and touch their elbows to the thighs. The number of correctly completed sit-ups performed in 30 s was the score recorded and used in data analysis^27^.

#### Flexibility

Sit-and-reach test was used to measure lower back and hamstring flexibility. To perform this test, the participants sat down on the ground before removing their shoes. One leg was extended, and the foot placed flat on the front end of the apparatus, while the knee of the other leg was bent with the foot flat on the floor and 5–8 cm to the side of the straight knee. The participants were instructed to slowly reach forward with extended arms along the measurement scale as far as possible, placing one arm on top of the other with hands facing palms down. Subjects were required to hold the position for at least 1 s. The participants were asked to repeat the test on two occasions and then to switch the position of their legs and repeat the reach for another two attempts. The best result of two trials of each side was used for the analysis^24^.

### Other measures

A standard stadiometer (Harpenden Stadiometer, Holtain Ltd) calibrated to the nearest 0.1 cm was used to measure height. Weight was measured at 0.1-cm intervals using a calibrated TANITA digital scale (TBF-410). Body mass index (BMI) was calculated by dividing the weight in kg by the square of height in cm (kg/m^2^). Participants’ MVPA levels during school, leisure time and transportation were collected before and after the intervention using a self-administered, modified Chinese version of the Global Physical Activity Questionnaire^19,28^. A combination of the parent- and teacher- proxy questionnaires was adopted to increase the accuracy of the responses considering that the participants may not be able to answer the questions independently. Demographic characteristics (age, sex and levels of ID) and comorbidities (Down’s syndrome, attention deficit/hyperactivity disorder and autism) were reported by participants’ parents/guardians in the baseline questionnaire. Process evaluation of the intervention including the attendance, intensity compliance rates and perceived intervention satisfaction, effectiveness and/or usefulness from the participants, parents/guardians and school teachers have been reported^19^.

### Statistical analysis

Descriptive statistics were used to characterize the study populations at baseline. An independent sample *t* test was used to measure the differences between groups at baseline, and between-group differences in changes in MVPA. The within-group changes in outcomes and MVPA over time were assessed using paired *t* tests. General linear models (GLMs) were applied to evaluate the between-group differences in changes in outcomes between pretest and posttest, adjusting for sex, comorbidities, and baseline outcome values. Statistical significance was considered as a p-value less than 0.05. All analyses were undertaken using R (Version 3.6.1).

## Results

**Figure 1:**
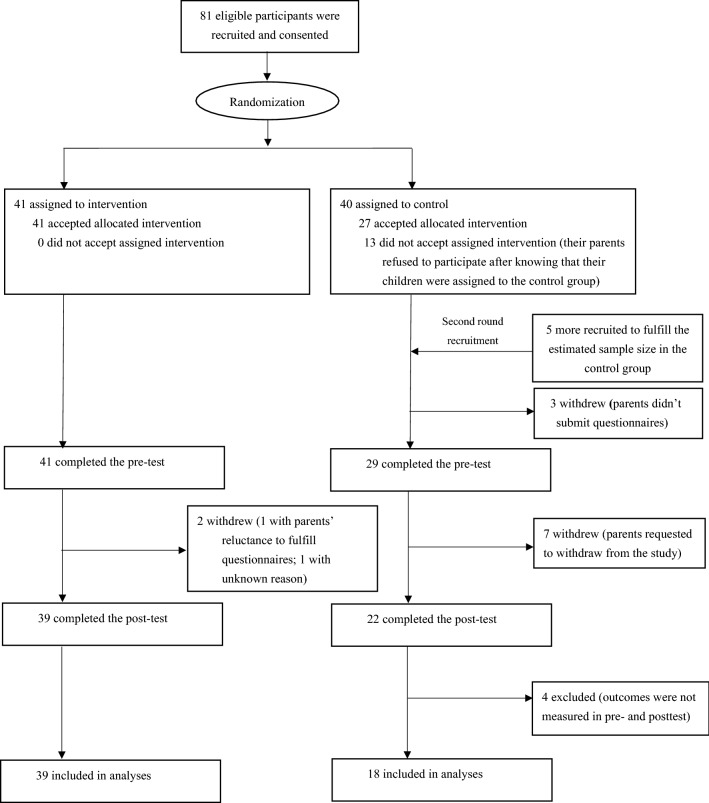
Flow diagram of participants in the study.

Participants in this study were recruited from June 2018 to July 2018. Of the 81 eligible participants who initially agreed to participate, 13 in the control group withdrew from the study after being informed of the random assignment results. Accordingly, to meet our sample size requirement (32 in each group^19^), five more participants were then recruited into the control group in the second-round recruitment. Out of the 61 participants (39 in the intervention group and 22 in the control group) who completed the entire study, 4 with missing outcome data in the control group were excluded from the analysis. Details of the participants’ flow diagram are shown in Fig. 1.

### Baseline characteristics

Table 1 summarizes baseline characteristics. The mean age of participants was 15.0 years (standard deviation 1.6 years), and 75.4% were boys. The majority of participants had autism (49.1%).

**Table 1:** Characteristics at baseline of study participants.

Variable	Total (N = 57)	Intervention group (n = 39)	Control group (n = 18)
	Mean (SD)	Mean (SD)	Mean (SD)
Age (years)	15.0 (1.6)	14.95 (1.5)	15.1 (1.8)
Height (cm)	162.8 (10.4)	163.1 (10.3)	162.1 (10.9)
Weight (kg)	74.6 (14.6)	75.5 (15.2)	72.7 (13.2)
BMI (kg/m^2^)	28.0 (3.8)	28.2 (3.7)	27.7 (4.1)
MVPA (min/day)	23.3 (18.3)	23.8 (19.5)	22.3 (15.7)

	*n* (%)	*n* (%)	*n* (%)
**Sex**			
Boys	43 (75.4)	31 (79.5)	12 (66.7)
Girls	14 (25.6)	8 (20.5)	6 (33.3)
**ID level**			
Moderate (IQ range 35–54)	28 (49.1)	16 (41.0)	12 (66.7)
Mild (IQ range 55–69)	29 (50.9)	23 (59.0)	6 (33.3)
**Comorbidities**			
Down syndrome	5 (8.8)	3 (5.1)	2 (16.7)
ADHD	17 (29.8)	15 (38.5)	2 (11.1)
Autism	28 (49.1)	24 (61.5)	4 (22.2)
**Number of comorbidities**			
0	18 (31.6)	6 (15.4)	12 (66.7)
1	29 (50.9)	25 (64.1)	4 (22.2)
2	10 (17.5)	8 (20.5)	2 (11.1)

SD, standard deviation; BMI, body mass index; MVPA, moderate-to-vigorous physical activity; ID, intellectual disability; IQ, intelligence quotient; ADHD, attention deficit/hyperactivity disorder.

### Changes in MVPA

The change in MVPA in the intervention group was 13.5 min/day [95% CI 7.69 to 19.34 min/day, *p* = 0.001] between pretest and posttest, whereas no significant changes were observed in the control group. A significant increase in MVPA was observed in the intervention group compared to controls (13.5 min/day vs − 2.4 min/day, *p* = 0.010).

### Effectiveness of intervention

The within-group changes and between-group changes in outcome measures were presented in Tables 2 and 3 respectively. The intervention group exhibited no significant changes in 9-min run/walk, whereas the control group experienced a significant reduction of 148.2 m ([95% CI − 284.10 to − 12.23], *p* = 0.034). These results led to a significant between-group difference in changes in 9-min run/walk from baseline to nine months (413.6 m, [95% CI 146.72 to 680.41], *p* = 0.003). Compared with baseline, although both groups improved their 30-s sit-ups (times) at 9 months with a mean of 2.5 (95% CI 1.31 to 3.69) and 1.5 (95% CI 0.35 to 2.71), respectively, the adjusted changes in the intervention group relative to the control group was not statistically significant. The scores for the sit-and-reach test were significantly improved in the intervention group by 1.7 cm (95% CI 0.53 cm to 2.81 cm) for the left leg and 2.8 cm (95% CI 1.76 cm to 3.78 cm) for the right leg (*p* < 0.01 in both cases, compared with the baseline), but not in the control group. After adjusting for sex, comorbidities, and the baseline values, the mean difference between the two groups in changes in sit-and-reach (right) was significantly different (2.2 cm [95% CI 0.37 to 4.09], *p* = 0.020), but no significant between-group difference was detected in left sit-and-reach. There were no significant within-group or between-group changes observed in handgrip strength (left and right).

**Table 2:** Within-group changes in outcomes from the pretest to the posttest in the study groups.

Outcome	Intervention (n = 39)			Control (n = 18)		
	Pretest Mean (SD)	Posttest	Mean difference (95% CI); *P*-value^a^	Pretest Mean (SD)	Posttest	Mean difference (95% CI); *P*-value^a^
9-min run/walk (m)	939.6 (431.4)	1094.0 (475.3)	154.4 (− 22.36 to 331.18); 0.085	942.2 (324.0)	794.1 (268.8)	− 148.2 (− 284.10 to -12.23); 0.034*
Handgrip strength, left (kg)	17.1 (7.2)	17.7 (6.5)	0.6 (− 1.04 to 2.30); 0.451	18.7 (7.9)	18.0 (7.0)	− 0.7 (− 3.77 to 2.33); 0.624
Handgrip strength, right (kg)	17.4 (6.8)	18.6 (6.1)	1.2 (− 0.12 to 2.57); 0.072	18.8 (8.0)	18.7 (6.1)	− 0.1 (− 2.51 to 2.23); 0.903
30-s sit-ups (times)	11.8 (5.0)	14.3 (5.3)	2.5 (1.31 to 3.69); < 0.001*	12.7 (5.4)	14.2 (5.7)	1.5 (0.35 to 2.71); 0.014*
Sit and reach, left (cm)	18.1 (8.6)	19.8 (9.0)	1.7 (0.53 to 2.81); 0.005*	20.1 (7.8)	20.3 (7.6)	0.2 (− 1.58 to 2.02); 0.798
Sit and reach, right (cm)	17.8 (8.4)	20.5 (8.5)	2.8 (1.76 to 3.78); < 0.001*	20.4 (8.69)	21.1 (8.2)	0.7 (− 0.40 to 1.73); 0.204

CI, Confidence interval.

*p < 0.05.

^a^Within-group difference was compared using paired sample *t* test.

**Table 3:** Between-group differences in changes in outcomes from the pretest to the posttest.

Outcome	Mean difference (95% CI)	*P*-value^a^
9-min run/walk (m)	413.6 (146.72 to 680.41)	0.003*
Handgrip strength, left (kg)	2.4 (− 0.66 to 5.36)	0.123
Handgrip strength, right (kg)	− 0.1 (− 2.56 to 2.38)	0.943
30-s sit-ups (times)	0.5 (− 1.71 to 2.76)	0.640
Sit-and-reach, left (cm)	1.3 (− 1.13 to 3.68)	0.290
Sit-and-reach, right (cm)	2.2 (0.37 to 4.09)	0.020*

CI, Confidence interval.

*p < 0.05.

^a^Between-group difference is the mean change difference of the intervention group relative to the control group, which was calculated by the general linear model with the intervention group as the main effect; sex, the presence of comorbidities and the baseline value of outcome measures as covariates.

## Discussion

We estimated the effects of a 9-month APA program on HRPF among 57 adolescents with ID who were overweight and obese. The results from the GLMs on between-group differences showed that this program significantly improved cardiorespiratory fitness (tested by 9-min run/walk) and potentially improved flexibility (tested by sit-and-reach). However, the between-group differences in changes in muscular strength and endurance (tested by handgrip strength and 30-s sit-ups) were nonsignificant. The program adopted an RCT design, had an overall moderate intensity, and was delivered at a frequency of twice a week with each session lasting for 45 min. Very few interventions have been focusing on improving HRPF for adolescents with ID, the findings from the study added to the limited knowledge base.

Cardiorespiratory fitness is considered an important indicator of cardiometabolic health. Poor levels of cardiorespiratory fitness in childhood are associated with cardiovascular diseases and all-cause of mortality in later life^29^. It has been reported increased PA is associated with improved levels of cardiorespiratory fitness^30,31^. The possible mechanism may be caused by the exercise-induced changes in oxygen delivery, cardiac, vascular, and blood tissues^32^. In typically developing adolescents, substantial studies showed that both moderate and vigorous exercises have a beneficial impact on cardiorespiratory fitness^33,34^ This evidence has also been supported in adolescents with ID. According to the findings of our systematic review and meta-analysis^18^, thirteen of sixteen studies reported significant improvements in cardiorespiratory fitness (tested by maximal oxygen uptake, HR-related outcomes, shuttle run performance, or six-minute walk distance test), with duration, frequency, and length of intervention ranged from 30 to 90 min/session, one to five sessions/week, eight weeks to two years, respectively. The majority of studies adopted aerobic and/or resistance exercise involving moderate and/or vigorous intensity. Three studies^35–37^ applied 6-min walk distance tests were included in the meta-analysis and indicated a mean pooled difference of 51.85 m in favor of the intervention group. The three studies performed PA interventions at moderate-to-vigorous intensity, two to three sessions per week, 50 to 60 min per session over a period of 10–15 weeks. In line with those studies of adolescents with and without ID, our study found significant improvements in cardiopulmonary fitness in adolescents with ID, indicated by a significant between-group effect on the 9-min run/walk distance.

Though the between-group difference in cardiorespiratory fitness was significant, we failed to observe a significant improvement within the intervention group. Thus, the significant result may be partially due to a decline in cardiorespiratory fitness in the control group. As reported somewhere else^19^, we observed a significant increase in BMI in the control group. Evidence has indicated that increased BMI may hinder the development of cardiorespiratory fitness^38^. It is therefore possible that the observed decline in cardiorespiratory fitness might be due to the increment in BMI. Collectively, this 9-month PA intervention is effective to control and reduce obesity and maintain the cardiorespiratory fitness level for overweight and obese adolescents with ID.

Adolescents in both the intervention and control groups increased their performance of 30-s sit-ups after nine months. Although the increase was greater among the intervention group, no significant difference between groups was found. This is probably inconsistent with findings from our systematic review^18^ and other studies^35,39,40^, in which statistically significant improvements in muscular endurance between groups were reported. For example, research conducted by Wu et al.^39^ applied vigorous aerobic and muscular endurance exercises (i.e., sit-ups and consecutive jumps), with five 50-min sessions per week, lasting for 10 weeks, and suggested significant improvements in sit-ups performance after the intervention both within groups and between groups. The other two studies by Xu et al.^40^ and Kong et al.^35^ adopted 16-week gymnastics exercise with three 50-min sessions per week, and 12-week aerobic dance with two 60-min sessions per week, presenting significant improvements in sit-ups performance in favor of the intervention group. The exercises performed in the above studies (e.g., fundamental movement skills training) involved core strength training which may contribute to the enhancement in abdominal muscular endurance. However, a meta-analysis was not conducted due to a high level of heterogeneity observed across studies^18^. The limited number of studies reported with insufficient details of exercise protocols and discussion of results, or low-quality study design (e.g., non-RCT, single-arm), therefore, it is difficult to compare them with our study and draw a firm conclusion. In addition, compared with our long-term intervention lasting for 9 months, most of the studies are short duration (i.e., lasting for 10–16 weeks), which probably contributes to the observed significant improvements between groups in 30-s sit-ups. We recommend future studies report exercise protocols in detail, which would facilitate exploring components of an exercise program that are effective to improve muscular strength and endurance for adolescents with ID.

The handgrip strength test is simple and widely used to reflect general muscular strength, which was adopted to indicate muscular strength in the present study. The lack of significant improvements in handgrip strength was consistent with previous studies^35^ and was not unexpected because both interventions did not include specific upper limb strength training. In contrast, interventions involving targeted muscle training had positive effects on handgrip strength. For example, Elmahgoub et al.’s study^41^ illustrated significantly positive effects of a 10-week training program on upper limb muscle strength tested by handgrip strength. Exercise protocol involved 10-min upper limb strength training exercise (eg. biceps brachii and triceps brachii) in each total 50-min session, engaging three times per week^42^. Though the handgrip strength is a good indicator of health-related strength, exercises targeted upper limb muscle strength involved were not enough, thus the handgrip strength test itself may not truly reflect improvements on other muscle groups (e.g., lower limb strength)^43^, which may underestimate the intervention effects on muscular strength. Other tests (e.g., sit-to-stand test) are suggested to indicate muscular strength and draw robust conclusions in the future studies. Given that promoting muscular strength and endurance is beneficial for adolescents’ motor skills, cardiovascular health and skeletal health^1^, more muscular strength exercises are suggested to add in future interventions for adolescents with ID.

Flexibility has long been considered a major component of physical fitness as it helps to reduce chances of injuries, back pain, and musculotendinous strains, it is beneficial in improving sports performance and balance^44^. Our study verifies that the APA program has a potentially positive impact on flexibility among adolescents with ID, by presenting significantly improved test scores of sit-and-reach on the right side compared to the control group. The improvements in flexibility on both sides were detected in the intervention group from pre- and post-test, although the between-group difference on the left side could not reach the statistically significant level. Few studies have yet to evaluate the effects of the intervention on flexibility among adolescents with ID. Our systematic review identified four studies^35,42,45,46^ included PA interventions that evaluated flexibility (tested by sit-and-reach), in which three of the studies adopted a quasi-experiment study design, and one used an RCT design. None of them have found significant intervention effects on flexibility. Previous studies suggested that targeted specifically flexibility exercise (e.g., stretching) could improve flexibility^46,47^. For example, an adapted rhythmic gymnastics intervention program incorporating various forms of limb stretching probably has contributed to the increased flexibility of muscles and spine^48^. In the current study, the potential improvement of flexibility might be mainly attributed to the stretching exercise during the warm-up and cool-down period^49^. However, the stretching exercise involved in warm-up and cool-down might be insufficient, additional targeted exercises (e.g., stretching, gymnastics, yoga) are highly suggested to improve flexibility in future interventions. In addition, given the relatively small sample size, there might not be sufficiently powered to detect the significant difference between groups. Therefore, more interventions involving targeted flexibility exercises with rigorous study design are urgently needed to facilitate improvements in flexibility for adolescents with ID^50^.

Our study has several strengths, including the RCT design, assessor blinding, standardized measurements, low attrition rates, and high attendance rates^19^. However, several limitations should be noted in this study. Firstly, we did not perform an intention-to-treat analysis, which may result in overestimating the effectiveness of the intervention. Whereas, due to the high compliance and attendance rates in this study, the overestimation could be minor. Secondly, we observed a higher dropout rate in the control group (32.5%), resulting in unequal sample sizes in the two parallel groups. This may decrease the statistical power. Thirdly, previous evidence has documented that physical fitness components were associated with and affected by hormonal alterations during puberty^51,52^. However, we failed to collect information on pubertal growth, because the school teachers strongly suggested not to involve these related questions in considering of parents/guardians would not be accepted such questions. As a result, we did not collect the information on puberty and its influence on the intervention effects observed could not be ruled out. Fourthly, we used %HRR to monitor the intensity of exercises as most of them involved aerobic exercises and lacked of specific resistance exercises. Repetitions maximum will be suggested to monitor the intensity of resistance exercises in future studies. Lastly, there was no follow-up in this study. Therefore, we cannot confirm whether the effectiveness could be sustained after the intervention.

In conclusion, this APA intervention was effective in improving cardiorespiratory fitness and flexibility, but not muscular strength and endurance among adolescents with ID and overweight/obesity. In addition, it is feasible and practicable to implement such school-based APA intervention for this special population. Future research with rigorous study designs, comprehensive components of physical fitness, larger sample size, and long-term duration is warranted to reach more conclusive results to improve other HRPF components than cardiorespiratory fitness, as well as long-term health among adolescents with ID.

## Supplementary Information

Supplementary Information.

## Data Availability

The datasets analyzed during the current study are available from the corresponding author on reasonable request.
