# Supplementary material for: Effectiveness of an adapted physical activity intervention on health-related physical fitness in adolescents with intellectual disability: a randomized controlled trial
Source: Sci Rep. 2022 Dec 30;12:22583. doi: 10.1038/s41598-022-26024-1 (PMC9803657; doi:10.1038/s41598-022-26024-1)
Supplement: Supplementary file 1 — Supplementary Information. [file 41598_2022_26024_MOESM1_ESM.docx]

Table S1. Description of the APA training program

| Stages  (time period) | Target intensity of exercise | Types of exercise (duration) | Detailed exercises | Rules and descriptions | Adjustments to meet target intensity |
| --- | --- | --- | --- | --- | --- |
| 1  (3 months) | 30-40% HRR | Warm-up (5 minutes) | - Stretching activities to music | - Warm up (whole body) through a series of simple stretching movements to music. | - Nil |
|  |  | Main-exercise (35 minutes) | - Obstacle walk/run (20 meters) - Shuttle walk/run to collect beanbags (20 meters) - Jumping jacks - High knees | - Obstacle run: put several traffic cones on the track, participants need to walk/run around the traffic cones until they reach the finish line. - Shuttle run to collect beanbags in a 20-metre distance, following “beeps” sent by instructors. - Jumping jacks: 10 seconds/set, 4 sets, with 1-minute break between sets. - High knees: 10 seconds/set, 4 sets, with 1-minute break between sets. | - Increasing/decreasing speed by switching between walking and running - Increasing/decreasing the number of cones to adjust speed. - Increasing/decreasing the intervals between “beeps”. - Increasing/decreasing repetitions/duration of each set and break. |
|  |  | Cool-down (5 minutes) | - Stretching | - Stretching of upper limbs, abdomen and lower limbs. | - Nil |
| 2  (3 months) | 40-50% HRR | Warm-up (5 minutes) | - Stretching activities to music | - Warm up (whole body) through a series of simple movements. The participants should try to follow the rhythm of the music. | - Nil |
|  |  | Main-exercise (35 minutes) | - Obstacle run (20 meters) - Shuttle run to collect softballs (20 meters) - High knees - Sit-ups | - Obstacle run: put several traffic cones on the track, participants need to run around the traffic cones until they reach the finish line. - Shuttle run to collect softballs in a 20-metre distance, following “beeps” sent by instructors. - High knees: 20 seconds/set, 4 sets, with 40-second break between sets. - Sit-ups: 20 seconds/set, 4 sets, with 40-second break between sets. | - Increasing/decreasing the number of cones to adjust speed. - Increasing/decreasing the intervals between “beeps”. - Increasing/decreasing repetitions/duration of each set and break. |
|  |  | Cool-down (5 minutes) | - Stretching | - Stretching of upper limbs, abdomen and lower limbs. | - Nil |
| 3  (3 months) | 50-60% HRR | Warm-up (5 minutes) | - Stretching activities to music | - Warm up (whole body) through a series of simple movements. The participants should try to follow the rhythm of the music. | - Nil |
|  |  | Main-exercise (35 minutes) | - Watch me (game) - Jump, jump, throw (game) - High knees - Sit-ups | - Watch me: dribbling and layup: Divide participants into several groups. Dribble the soft volleyball/basketball and walk/run around the traffic cones (a 10-metre distance). After arriving at the end point, throw the ball into the basket. The participant needs to start again if the ball rolls away. - Jump, jump, throw: Six to eight participants in a group. Following the tutor’s instructions (e.g., single-leg jump, two-leg jump or jumping jack), the participants jump across five colour discs/ hula hoops, run to the throw line, pick up a bean bag and throw it into a hula hoop/ basket (5 metres distance). Then, they jump across the five colour discs/ hula hoops back to the start point. The next team member then repeats the tasks. - High knees: 30 seconds/set, 4 sets, with 40-second break between sets. - Sit-ups: 30 seconds/set, 4 sets, with 40-second break between sets | - Increasing/decreasing the distance. - Increasing/decreasing the time spent in each end line. - Increasing/decreasing group numbers to decrease/increase the waiting time. - Increasing/decreasing repetitions/duration of each set and break. |
|  |  | Cool-down (5 minutes) | - Stretching | - Stretching of upper limbs, abdomen and lower limbs. | - Nil |

HRR: Heart rate reserve.
